# Treatment of Proximate Surfaces

**Published:** 1891-06

**Authors:** W. H. Dwinelle


### TREATMENT OF PROXIMATE SURFACES.[1]

BY W. H. DWINELLE, M.D., D.D.S.

I HAD thought that this subject of contours had been exhausted long ago. It has been a constant theme with me, and one which I have advocated for nearly forty years. Dr. Perry's admirable essay on the treatment of proximal surfaces, read before the Odontological Society of Pennsylvania, seemed to make it impossible for anything further to be said on the subject, but by Dr. Wilson's paper we have been treated to an entertainment pertaining to the matter so fresh, original, and in many ways so new that we are almost ready to question whether the subject had been thoroughly discussed after all.

I have so identified myself with the subject of the paper, and advocated its theory and practice for so long a period, with more or less opposition, that I take pleasure in thanking the essayist for his able and admirable reminder of principles which reach back to the teachings of nature herself.

And here I will put in a plea for nature. From time immemorial it has been the highest aim of art to imitate nature. She has ever been the great example which we may continually approach yet never reach; and those productions will ever be considered most perfect which most nearly resemble her own.

The wisdom of contouring teeth to their original form seems to me to be one of those self-evident propositions which is beyond all question. It ought to be quite sufficient for us to know that it has the seal and approval of the Divine architect himself, who created nothing in vain.

The uses of the contour forms of the teeth and the mechanical principles involved in them we may partially enumerate, but it is foolhardy for us to assume that we can comprehend them all. We have no right to impeach the wisdom of the Almighty and limit His infinity down to our poor finiteness. We have no right to repudiate and reject His sample copy given to us for imitation; it becomes, in a certain sense, sacrilegious for us to do so, for it assumes that ours is superior to the source and embodiment of all wisdom, whose knowledge and experience antedates eternity itself.

I might dilate on the beauty and wisdom displayed in the for-

---

[1] Read before the New York Odontological Society, March, 1891.

mation of the two dental arches, of which a single tooth, with its many qualities and functions, is an integral part, but I forbear.

To understand the laws of nature and her plans so far as possible, and accept and apply them, is our first duty.

When you have restored opposing surfaces of teeth to their original contours, so that their approximal sides meet at their largest diameter, taking care to faithfully close all the *joints* of their walls down to the very surface of the enamel and Nasmyth's membrane, which, as you know, is akin to fluor-spar, whose integrity fluoric acid alone can impair, you have virtually restored it to all its functions as of old and secured it in its position in the arch, and have done all that art can do and reached the highest approximation to nature. It seems to me to be folly for any one to question the wisdom of this course and advocate in its place the mutilation of the teeth and flat fillings with exposed enamel and dentine borders, together with the mechanical and physical disadvantages that always follow operations of this character. It has been objected that teeth when contoured become thereby frail and are easily broken down and destroyed. In a treatise I wrote on this subject in 1855, when contour fillings were first advocated, I demonstrated that, by proper anchorage and undercutting, the teeth are so locked and banded together that the crown becomes stronger than by treating by the old methods. In case of treating a tooth that has lost its vitality, the pulp-cavity can be so utilized that a broad column of gold can be built up from its centre that will be sufficient to enable the contour to resist any force it might meet with.

I frequently see contour fillings made in this city more than thirty years ago which are as perfect to-day at their cervical borders and in all their appointments as though they had been completed but yesterday. Many of these have been seen and approved of by gentlemen present.[1]

I cannot approve of the practice of slicing off a quarter or third of the enamel and dentine from a tooth and leaving the remainder to the possibilities of a flat filling. When we consider the construction, economy of, and the relation to, and position of the enamel rods, it would seem like displacing the key-stone of a succession of arches, thereby endangering the entire superstructure.

---

[1] I have advocated the practice of contour fillings for nearly forty years, and my daily and constant observation in the practice of others continually confirms me in the faith.

To those who contend that flat fillings are superior to the contour, which restores the tooth to its original form and function, I would simply say that they assume that a fraction of a tooth is superior to the original tooth itself. Any deviation from the strict construction of the *contour* must be on the ground of *expediency*, which we admit qualifies every operation that passes through our hands, but that a duplication of nature by art is superior to a partial success in that direction, it seems to me, is admitted by all.

I could refer to some of the objections to flat fillings, among others that all normal articulation is virtually broken up, thereby bringing with them a train of evils apparent to every one, so that teeth thus treated lose their character as teeth in a large sense, but I forbear.

The discussion of this subject is suggestive of the old inverted aphorism that "a part is greater than the whole!" I think I cannot better conclude this brief article than by quoting, *first*, from Dr. Perry's paper, already alluded to, where, after reviewing the subject of contours in a manner both exhaustive and entertaining, he summarizes the whole by saying, "Get free edges, if possible, for your approximal fillings, and shape them to the original outline of the teeth;" and, *secondly*, from Dr. Allan's very able paper, read before the annual meeting of the First District Society, January, 1890. In commenting on this afterwards, Dr. Allan said, "I am sorry my meaning has been lost. Read between the lines.

"I intended my paper to be a strong argument in favor of contour work. I am ready to say that, where all indications are favorable, the contour work is both theoretically and practically the best."

This is all we contend for, and admits the whole question.

If not practical and inexpedient, do not do it. If practical and expedient, *do it!*

